# Supplementary material for: A set of multi-entry identification keys to African frugivorous flies (Diptera, Tephritidae)
Source: Zookeys. 2014 Jul 24;(428):97–108. doi: 10.3897/zookeys.428.7366 (PMC4143993; doi:10.3897/zookeys.428.7366)
Supplement: Supplementary material 4 — Key to Capparimyia [file zookeys-428-097-s004.zip › SF4_ZooKeys_key to Capparimyia/lucid_player/help/help/system_requirements.htm]

System Requirements


# System Requirements

#### Java Virtual Machine v1.4.2 or greater

#### Windows OS

98/ME/NT(SP6a)/2000(SP3)/XP(SP1)/2003  
Pentium 166MHz  
HD: 75MB  
RAM: 32MB

#### Macintosh OS

OS X 10.3 or greater  
HD: 51MB  
RAM: 128MB

#### Linux OS

A flavour of Linux that supports J2RE, such as: Red Hat 7.3/8.0 SuSE
8.0, TurboLinux 7.0 or SLEC 8   
Pentium 166MHz  
HD: 85MB  
RAM: 32MB

#### Solaris OS: 7, 8, 9 or 10

RAM: 128MB.  
HD: Up to 70MB
